# CCNDBP1, a Prognostic Marker Regulated by DNA Methylation, Inhibits Aggressive Behavior in Dedifferentiated Liposarcoma *via* Repressing Epithelial Mesenchymal Transition

**DOI:** 10.3389/fonc.2021.687012

**Published:** 2021-09-22

**Authors:** Lingge Yang, Zhiqiang Wu, Wei Sun, Peng Luo, Shiqi Chen, Yong Chen, Wangjun Yan, Yan Li, Chunmeng Wang

**Affiliations:** ^1^Department of Musculoskeletal Oncology, Fudan University Shanghai Cancer Center, Shanghai, China; ^2^Department of Oncology, Shanghai Medical College, Fudan University, Shanghai, China; ^3^Fudan University Shanghai Cancer Center, Key Laboratory of Medical Epigenetics and Metabolism, Institutes of Biomedical Sciences, Fudan University, Shanghai, China

**Keywords:** dedifferentiated liposarcoma, CCNDBP1, prognosis prediction, invasion and metastasis, epithelial-mesenchymal transition, DNA methylation

## Abstract

The present study aimed to explore the prognostic value, function, and mechanism of CCNDBP1 in dedifferentiated liposarcoma (DDL). Immunohistochemistry staining was used to analyze the protein expression of CCNDBP1 in tissue specimens. After silencing CCNDBP1 in LPS853 and overexpressing CCNDBP1 in LPS510, CCK-8, clone formation, transwell migration, and invasion assays were used to detect cell proliferation, migration, and invasion ability. CCNDBP1-induced cell apoptosis was analyzed by flow cytometry. The altered expression of epithelial-mesenchymal transition (EMT)-related proteins were detected by Western blot. The methylation, gene expression, and clinical data of 58 samples with DDL were analyzed using the cancer genome atlas (TCGA) database. Low expression of CCNDBP1 was associated with a poor prognosis of patients with DDL and was considered an independent prognostic factor of the progression-free survival (PFS). CCNDBP1 significantly inhibited the clone formation, proliferation, migration, and invasion of cancer cells *in vitro* and promoted cancer cell apoptosis. CCNDBP1 could repress the pathological EMT, thereby inhibiting the malignant behaviors of DDL cells. The high degree of DNA methylation sites cg05194114 and cg22184989 could decrease the expression of CCNDBP1 and worsen the prognosis of DDL patients. This is the first study reporting that *CCNDBP1* is a tumor suppressor gene of DDL and putative prognostic marker in DDL patients. CCNDBP1 might inhibit the ability of cell proliferation and invasion by repressing pathological EMT, and the expression of CCNDBP1 could be regulated by DNA methylation in DDL.

## Introduction

Liposarcoma is a mesenchymal malignancy with distinct tissue diversity, consisting of adipocytes with different degrees of differentiation and heteromorphosis. Well-differentiated liposarcoma (WDL) and dedifferentiated liposarcoma (DDL) are the most common types of liposarcoma, accounting for about 40%–45% of the liposarcomas (1). The invasion ability of WDL is poor; it does not appear metastatic but has the tendency of recurrence (2). In addition, it could be dedifferentiated and converted into DDL, obtaining a stronger invasive ability, rapid local recurrence, and distant metastasis, which occurs in about 10% of WDLs (3, 4). Based on the fifth Edition of the WHO Classification of Soft Tissue and Bone Tumors, both DDL and WDL are characterized by supernumerary ring chromosomes and/or giant marker chromosomes composed of amplified products from the q13-15 region on chromosome 12 (2). Therefore, DDL shares the amplification of *MDM2* and *CDK4* with WDL, while additional abnormalities are frequently found in DDL than WDL, including the amplification of *JUN*, *MAP3K5*, *TERT*, and *CPM*, and deletion of *ATRX*, *ATM*, *CHEK1*, *ABTB16*, *PPP2R1B*, and *EI24* (5).

Clinically, the location of WDL/DDL is the largest predictor of recurrence, the largest prognostic indicator, and the major adverse prognostic factor of DDL described as retroperitoneal location, where the tumor shows a worse survival rate than other locations (6). The predominant treatment of WDL/DDL is still surgery. Due to the poor sensitivity of WDL/DDL to radiotherapy and chemotherapy, patients generally do not receive adjuvant treatment after surgery. The local recurrence rate of DDL after the first operation was up to 20%, and 30% of the recurrent cases could develop tumor metastasis within 3 years, while the recurrence rate after the second operation was up to 44% (7). For advanced patients who could not undergo the operation, only a few systemic treatments are available. In the National Comprehensive Cancer Network (NCCN) guidelines of soft tissue sarcoma version 2.2021, only palbociclib is recommended for the systemic treatment of WDL/DDL. In the clinical trial report, the estimated 12-week progression-free survival (PFS) rate was 66% [90% confidence interval (CI): 51% to 100%], the median PFS was 17.9 weeks and one case (3%) achieved partial response (PR) (8). However, this drug is utilized only for the treatment of patients with advanced CDK4-amplified WDL/DDL. Therefore, effective biomarkers to predict recurrence and metastasis are an urgent need to open up new therapeutic routes for WDL/DDL.

CCNDBP1 (cyclin D1 binding protein 1) is also called DIP1, HHM, or Maid, and is a member of the dominant-negative helix-loop-helix (dnHLH) protein family and has the leucine zipper structure, with a molecular weight of 40 kDa. The CCNDBP1 functions by inhibiting Cyclin D1/CDK4 from preventing RB1 phosphorylation and blocking the dependent transcription of E2F, which negatively regulates the cell cycle process (9). Previous studies have shown that many proteins, such as diabetes-related Ras (Rad) (10), ribosomal phosphoprotein P0 (RPLP0) (11), and oligodendrocyte transcription factor 1 (Olig 1) (12), interacted with CCNDBP1, but the physiological functions were still unclear.

In this study, four tissue samples of DDL and the corresponding matched WDL were selected for high-throughput RNA sequencing, and overlapping genes with a differential expression were screened. In addition, genes with a prognostic value were obtained by analyzing 58 cases of DDL in the cancer genome atlas (TCGA) database (https://cancergenome.nih.gov) (13). Based on the intersection, the obtained prognostic value genes were verified on the sarcoma through the gene expression profiling interactive analysis (GEPIA) database (http://gepia.cancer-pku.cn) (14). Finally, *CCNDBP1* was identified as the research gene with a prognostic value in DDL. The prognostic value, molecular function, and mechanism of CCNDBP1 in DDL are investigated further.

## Methods

### Human Specimens and Cell Lines

All tissue specimens, including 8 cases of cancer tissues for sequencing analysis, 46 cancer specimens for immunohistochemical analysis, and 12 adjacent normal tissues (ANTs), were obtained from the Tissue Bank of Fudan University Shanghai Cancer Center. The surgical excision specimens. After being taken off, tissue specimens were sliced into small pieces of about 0.5 cm and mixed with 1 ml of RNAlater^®^, placed at 4°C overnight, and stored at -80°C for future use. The access to all tissue specimens was approved by the Institutional Review Board (IRB) of our cancer center (IRB number: 050432-4-1911D), and all were diagnosed pathologically. The tissue specimens were also detected by fluorescence *in situ* hybridization (FISH), and the results demonstrated *MDM2* amplification. Informed signed consent was obtained from the patients. The clinicopathological classification and staging were determined according to the criteria of the American Joint Committee on Cancer (AJCC) Eighth Edition.

LPS853 and LPS510 cell lines were a generous gift from Professor Yuexiang Wang of Shanghai Institute of Nutrition and Health, Chinese Academy of Sciences (Shanghai Branch) and Professor Jonathan A. Fletcher of the Department of Pathology, Brigham and Women’s Hospital, Harvard Medical School. The cells were cultured in a DMEM culture medium (Gibco, USA) containing 10% fetal bovine serum at 37°C and 5% CO_2_.

### RNA Extraction and RNA-Seq

TRIzol reagent (Invitrogen, USA) was used to extract RNA according to the instructions of the manufacturer. The VAHTSTM**^®^** Total RNA-seq (H/M/R) Library Prep Kit for Illumina^®^ was utilized to construct the transcriptome libraries of acceptable quality to the Annoroad company for sequencing on Illumina PE150 as the sequencing platform and with 10 Gb of clean data as the sequencing depth.

### Immunohistochemistry Staining of Tissue Specimens

The tissue specimens were paraffin-embedded, baked at 65°C for 30 min, then placed in 3% H_2_O_2_ to eliminate the activity of endogenous peroxidase after dewaxing, hydration, and antigen retrieval. Then, the CCNDBP1 antibody (Proteintech, USA, dilution rate: 1:50) was incubated with the slices for 1 hour in the wet box at room temperature, followed by IgG H&L (HRP, abcam, UK, dilution rate: 1:400) incubation of 30 min. After color development and baking the tissue at 65°C for 15 min, hematoxylin-eosin (HE) staining, xylene transparency, and neutral balsam mounting, the images were captured under the microscope for sample analysis. Image J software (15) was used to measure the grayscale of the images, and the average optical density (AOD) value of each image was obtained and linked to the clinicopathological features and prognosis information of the patients.

### Plasmid, Primers, and Small Interfering RNA

For the construction of pcDNA3.1-CCNDBP1, the full-length cDNA of *CCNDBP1* was obtained by quantitative polymerase chain reaction (qPCR) using gene-specific primers and subcloned between restriction endonucleases *XhoI-HF* and *BamHI-HF* of the pcDNA3.1 vector. The plasmids were transfected using Lipofectamine 3000 reagent (Invitrogen, USA) according to the instructions of the manufacturer. Primers, siRNAs, and plasmids used in experiments were synthesized by Asia Vector Biotechnology Company. The primers are listed in **Table S1**.

### Quantitative Reverse Transcription Polymerase Chain Reaction

After RNA extraction, the first-strand cDNA synthesis kit (TransGen, China) was used for the reverse transcription of RNA into cDNA. The qPCR reaction mixture (10 µl of SybrGreen qPCR Master Mix + 0.4 µl of upstream primer + 0.4 µl of downstream primer + 7.2 ul of ddH2O + 2 µl of cDNA, 20 µl in total) consisted of the SG Fast qPCR Master Mix (Sangon Biotech, China). The PCR reaction was as follows: Melting, 95°C for 7 s; Annealing, 57°C for 10 s; Extension, 72°C for 15 s; for 45 cycles. The relative expression of *CCNDBP1* was assessed according to 2^-ΔΔCT^, with the Ct value of *GAPDH* as the reference.

### Western Blot

The cells were lysed in RIPA lysate (Beyotime, China), and the protein was quantified used the BCA protein assay kit (Beyotime, China). An equivalent of 50 μg of protein sample was resolved by SDS-PAGE electrophoresis, and transferred to the membrane. The membrane was blocked and probed with CCNDBP1 (Proteintech, USA, dilution rate: 1:1,000) or GAPDH (CST, USA, dilution rate: 1:20,000) antibody overnight at 4°C, followed by IgG H&L (HRP, abcam, UK, dilution rate: 1:5,000) for 1 h at 37°C. The immunoreactive bands were observed *via* the ECL color appearance system (Thermo, USA) and had gray scale analysis. The Epithelial-Mesenchymal Transition (EMT) Antibody Sampler Kit (CST, USA) was used for the detection of EMT-related proteins.

### Cell Transfection

LPS853 cells were collected in the logarithmic phase and seeded in 12-well (5 × 10^5^ cells per well) or 96-well (5 × 10^3^ cells per well) plate. After complete cell adherence, CCNDB1-siRNA and the negative control were transfected, respectively, using Lipofectamine™ 2000 (Life Technologies, USA) for 6 h at the final concentration of 100 nmol/L and temperature 37°C. The transfection liquid was replaced with a DMEM complete medium containing 10% serum for continuous culture.

### Cell Clone Formation and CCK-8 Assays

Cell clone formation assay: The cells were transfected in 6-well plates (500 cells per well) and fixed with 4% paraformaldehyde. The cells were stained with crystal violet, and the number of clones was counted as >50 cells/group.

CCK-8 assay: After transfection and other treatments in a 96-well plate (about 1,500 cells per well, 3 parallel wells), a volume of 10 μl of CCK-8 (BBI Life Sciences, China) was mixed with 90 μl of culture medium at 0, 24, 48, 72, and 96 h after transfection, and incubated for an additional 2 h at 37°C. A microplate spectrophotometer (Biotek, USA) was utilized, and the absorbance was measured at 450 nm.

### Transwell Assays

Transwell migration assay: Non-serum DMEM culture medium was replaced for 24 h and incubated for an additional 24 h. The cells were collected on day 2, rinsed with phosphate-buffered saline (PBS) three times, resuspended in a non-serum DMEM culture medium, and the cell density adjusted to 2.0 × 10^5^ cells/ml, subsequently, 200 μl of the cell suspension was added into each Transwell chamber. The 24-well plate was placed into the Transwell chamber after adding about 700 μl of complete medium in the 24-well plate and cultured at 37°C. Subsequently, it was removed from the chamber after 24 h, and the culture medium was replaced with PBS containing 0.1% crystal violet and 10% methanol for fixing and dyeing for 30 min. Then, the chamber was placed in a 24-well plate and placed in an oven at 56°C for 2 h; the images were captured under a microscope.

Transwell invasion assay: The Matrigel-coated chamber was used, and the assay was performed as described in the Transwell migration assay.

### Cell Apoptosis Assay

The cells were trypsinized and suspended, followed by centrifugation at 1,500 rpm for 5 min. Subsequently, the cells were collected, mixed with propidium Iodide (PI) and Annexin V Staining Kit (Sangon, China), incubated at room temperature in the dark for 15 min, and detected by flow cytometry (Beckman Coulter, USA).

### Bioinformatics Analysis

Prognosis gene screening: We downloaded and integrated the transcriptome data (HTSeq-FPKM) of 58 DDL cases and the clinical data of the patients from the TCGA database. The survival pack of R 3.6.0 was used to divide the data into high-expression and low-expression groups according to the median of gene expression; the survival analysis was performed on the downloaded data. The ENSG-ID was converted into the official gene symbol *via* the Biomart database (http://asia.ensembl.org/biomart/martview) (16). The prognosis values of these genes were verified through the GEPIA database after obtaining the prognosis-related genes of the DDL patients.

Hallmark analysis: Gene set enrichment analysis (GSEA) for genes presented significant changes in the expression levels after cell transfection to identify the hallmarks of CCNDBP1 that were involved in regulation.

Selection of DNA methylation regulation sites: MethSurv database (https://biit.cs.ut.ee/methsurv/) (17) was used for the survival analysis of all the DNA methylation positions of CCNDBP1 in sarcoma patients, which were screened out based on the prognosis values. Then, the DNA methylation data (Illumina Human Methylation 450) of 58 DDL patients and clinical data from the TCGA database were downloaded and integrated. The linear regression of the correlation between CCNDBP1 expression levels and the methylation degrees were analyzed. These positions were related to the prognosis of the patients.

### Statistical Analysis

All experiments were repeated three times. IBM SPSS Statistics Version 25.0 and GraphPad Prism Version 8 were utilized for the statistical analysis of the experimental data. The differences in the measurement data were evaluated by Student’s t-test, and the enumeration data were assessed *via* chi-square test between the two groups; the differences among multiple groups were examined by variance analysis. The Kaplan–Meier method was used for survival analysis, and the difference in the sub-groups was compared *via* log-rank test. The significant prognosis factors were determined through a stepwise multiple Cox regression analysis. Only the prognostic factors with a statistical significance in the univariate analysis were included in the multivariate analysis. The data were considered significant with P < 0.05 (*), P < 0.01 (**), P < 0.001 (***), and P < 0.0001 (****) and expressed as mean ± standard deviation (SD) unless otherwise indicated.

## Results

### *CCNDBP1* Was a Differential Gene With a Prognostic Value Between Well-Differentiated Liposarcoma and Dedifferentiated Liposarcoma

**Figure 1:**
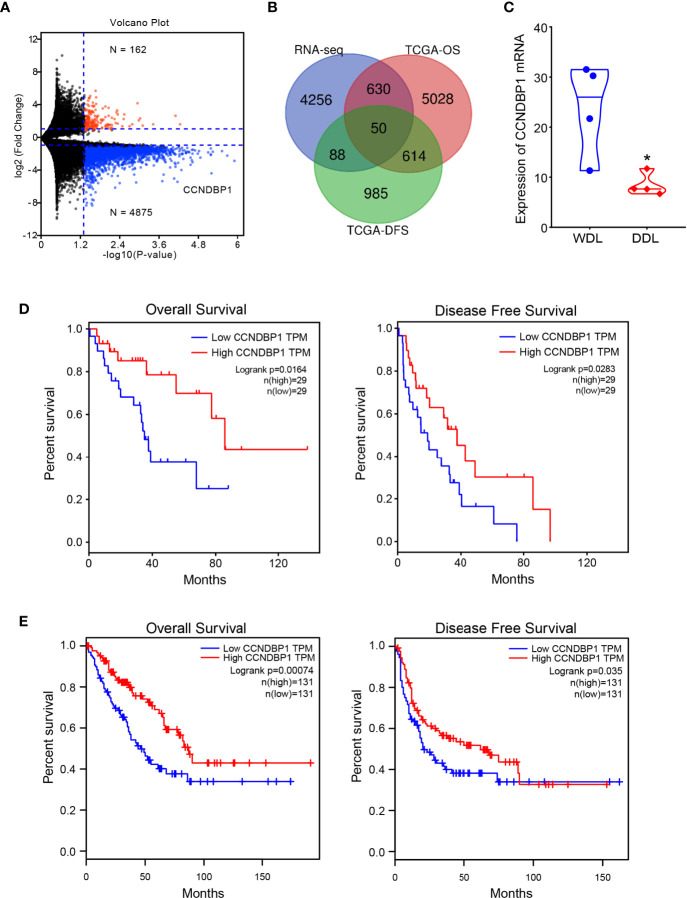
CCNDBP1 was a differential gene with a prognostic value between WDL and DDL. **(A)** Differential genes detected by high-throughput sequencing, red indicates upregulated genes and blue indicates downregulated genes in DDL compared with WDL; **(B)** Venn diagram, 50 genes that have expression differences between tissues of WDL and DDL, and have predictive value for the prognosis of patients; **(C)** the mRNA expression of CCNDBP1 had a significant difference between WDL and DDL tissues; **(D)** The expression level of CCNDBP1 in 58 patients with DDL in the TCGA database was correlated with the prognosis of the patients and the higher the expression level of CCNDBP1, the better the prognosis of patients; **(E)** The GEPIA database verified that the low expression of CCNDBP1 was a poor prognostic factor in patients with sarcoma. *P < 0.05.

The clinical baseline characteristics of the eight samples for high-throughput sequencing are shown in **Table S2**, and the results of RNA extraction concentration are shown in **Table S3**. After high-throughput sequencing, the differential genes were screened with |log_2_ fold change (FC)| > 1, and a total of 5,037 genes with statistically significant expression differences (P < 0.05) were identified, among which, 162 genes were upregulated, and 4,875 genes were downregulated in DDL compared with WDL (**Figure 1A**). In addition, the data of 58 cases of DDL in the TCGA database were analyzed. According to the median value of gene expression, the patients were divided into high- and low-expression groups, of which, 6,408 genes affected the overall survival (OS) of the patients and 1,737 genes affecting the disease-free survival (DFS) of the patients. Based on the integration of these genes, we obtained 50 genes with differences between the tissues of WDL and DDL, and have a predictive value for the prognosis of patients (**Figure 1B**). Next, these genes were assessed with respect to the sarcoma by the GEPIA database. Among these genes, only *CCNDBP1* and *VPS18* affect both the OS and DFS in patients with sarcoma, and the FC of *CCNDBP1* is higher than that of *VSP1*8 in our sequencing data. Finally, *CCNDBP1* was identified as the research gene with a prognostic value and significant differences in the mRNA expression of tissue samples between WDL and DDL (**Figure 1C**). CCNDBP1 had an improved prognostic value in 58 patients with DDL in the TCGA database (**Figure 1D**) and all the sarcoma patients in the GEIPA database (**Figure 1E**). Specifically, the higher the expression of CCNDBP1, the better the prognosis of patients.

### The Expression and Prognosis of CCNDBP1 in Dedifferentiated Liposarcoma

**Figure 2:**
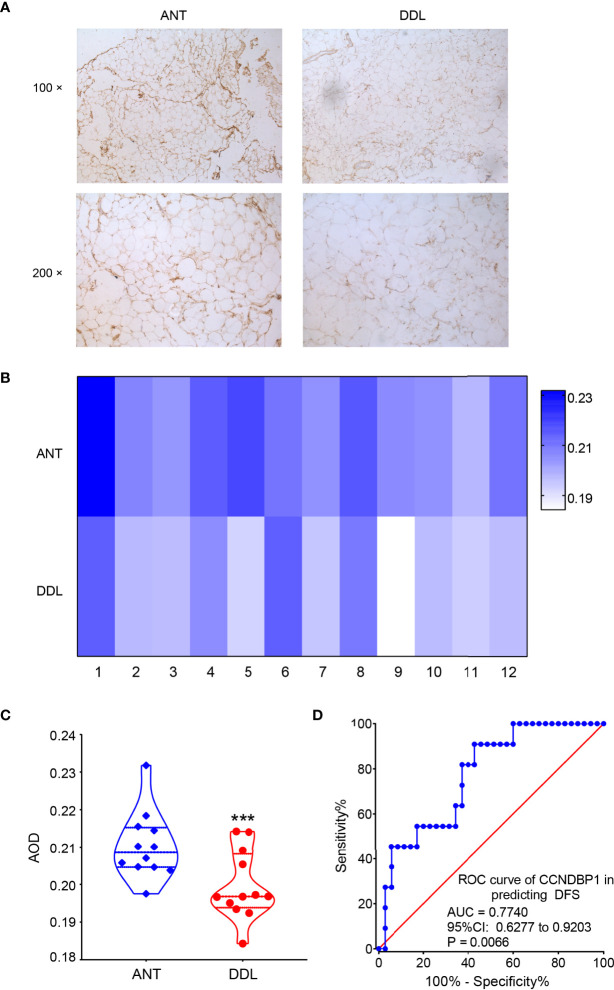
The expression of CCNDBP1 in tissues of DDL was significantly lower than that in the ANT. **(A)** The representative images of IHC staining of DDL and its ANT; **(B)** The heatmap of the AOD value of CCNDBP1 in 12 cases of DDL and the corresponding ANT; **(C)** The difference of CCNDBP1expression in DDL and their ANT was statistically significant; **(D)** ROC curve plotted based on the AOD value of IHC staining of CCNDBP1 and the PFS of the patients. ANT, adjacent normal tissue; ***P < 0.001.

We detected the expression of CCNDBP1 in 46 DDL samples and 12 corresponding adjacent normal tissues (ANTs) by immunohistochemistry (IHC). We found the expression of CCNDBP1 in tissues of DDL was significantly lower than that in the ANT (**Figure 2A**).

The IHC images were analyzed based on the gray intensity, and the AOD value of each image was obtained. Then, the expression levels of CCNDBP1 in the 12 cases of DDL and their corresponding ANT were analyzed according to the AOD value (**Figure 2B**), albeit with statistically significant differences (**Figure 2C**, P = 0.0007). Therefore, the expression levels of CCNDBP1 in DDL were lower than those in their ANT.

Next, we plotted the receiver operating characteristic (ROC) curve for predicting the PFS and OS of the patients using the AOD value of CCNDBP1 according to the prognosis of the 46 patients with DDL. The AOD value > 0.1950 had a certain predictive value for the PFS of patients [**Figure 2D**, area under the curve (AUC) = 0.7740, P = 0.0066], and the sensitivity and specificity were 90.91% and 57.14%, respectively. However, the predictive value for the OS of the patients was not satisfactory (AUC = 0.5852, P = 0.3224).

### Correlation Between the Expression of CCNDBP1 and the Clinicopathological Characteristics of Patients

This group of 46 patients, aged 37–87 (median: 57)-years-old, consisted of equal number of males and females. In the cohort, all patients underwent surgical treatment. Among them, 23 patients (50%) had primary DDL and they underwent surgery in our hospital for the first time (operation time = 1), while 23 patients (50.0%) had a recurrent DDL who underwent surgery before coming to our hospital and again due to recurrence (operation time ≥ 2). The tumor size ranged from 3 to 42 cm, and the median size was 11.8 cm. The tumors occurred in the trunk in 5 cases (10.8%), limbs in 7 cases (15.2%), and retroperitoneum in 34 cases (74.0%). The clinical staging was based on the AJCC Eighth Edition, with 5 patients (10.8%) at stage II, 14 patients (30.4%) at stage IIIA, 16 patients (34.7%) at stage IIIB, and 11 patients (23.9%) at stage IV. Among the metastatic DDL patients, 4 cases (8.7%) had lymphatic metastasis and 8 cases (17.4%) had distant metastasis; the metastatic sites included the lung, bone, large intestine, and spleen. Except for these 8 patients with distant metastasis, all the other patients underwent complete resection of the tumor. For histological grading, 17 cases (37.0%) were at G2 and 29 cases (63.0%) were at G3. The baseline information is summarized in **Table 1**.

**Table 1:** Baseline characteristics of the Patients (N = 46).

Characteristics	No. of patients	% of total
**Gender**		
Male	23	50.0
Female	23	50.0
**Median age (years, range)**	58	37–87
**Tumor size (cm, range)**	11.8	3–42
**Tumor site**		
Trunk	5	10.8
Limb	7	15.2
Retroperitoneum	34	74.0
**Operation times**		
1 time	23	50.0
≥2 times	23	50.0
**AJCC anatomic stage**		
Stage II	5	10.8
Stage IIIA	14	30.4
Stage IIIB	16	34.7
Stage IV	11	23.9
**T classification**		
T1	5	10.8
T2	14	30.4
T3	9	19.6
T4	18	39.2
**N classification**		
N0	42	91.3
N1	4	8.7
**M classification**		
M0	38	82.6
M1	8	17.4
**Histologic grade**		
G2	17	37.0
G3	29	63.0
**Vistal States (at follow-up)**		
Alive	22	47.8
Dead	24	52.2
Recurrence/metastasis	35	76.1
No recurrence/metastasis	11	23.9

All patients were followed-up by telephone and outpatient service at regular intervals (3–6-month interval postoperatively in 2 years and annually thereafter), and the follow-up ended on April 14, 2019. The follow-up time was 4.1–107.3 months and the median follow-up was 66.6 months. A total of 35 cases (76.1%) had recurrent or metastatic events, and the median PFS was 27.8 months (95%CI: 10.9–44.8 months). The 1-, 3-, and 5- PFS rates were 65.3%, 34.0%, and 17.0%, respectively. A total of 22 cases (47.8%) of patients survived, 24 cases (52.2%) died, and the median OS was 38.7 months (95%CI: 19.0–58.4 months). The 1-, 3-, and 5-year OS rates were 95.0%, 52.5%, and 39.5%, respectively.

**Figure 3:**
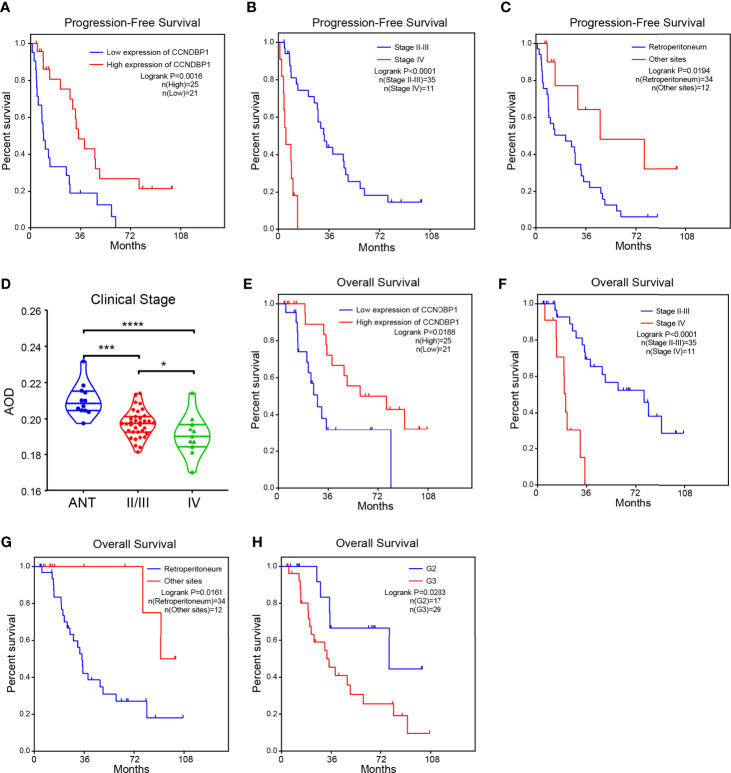
The higher the expression of CCNDBP1, the better the prognosis of patients. **(A)** Influence of CCNDBP1 protein expression level on the PFS of patients. The AOD value of 0.1950 as the boundary, the higher the CCNDBP1 protein expression level, the longer the PFS; **(B)** Influence of clinical staging on the PFS of patients. The later the clinical staging, the shorter the PFS; **(C)** Influence of tumor sites on the PFS of patients. The PFS of tumors located in the retroperitoneum is shorter than that of the other sites; **(D)** Relationship between CCNDBP1 protein expression level and the clinical staging of patients; the later the clinical staging level, the lower the CCNDBP1 protein expression level, and the protein expression of CCNDBP1 in ANT was significantly higher than that in sarcoma tissues; **(E)** Influence of CCNDBP1 protein expression level on the OS of patients; the higher the CCNDBP1 protein expression level, the longer the OS; **(F)** Influence of clinical staging on the OS of patients; the later the clinical staging level, the shorter the OS; **(G)** Influence of tumor sites on the OS of patients. The OS of tumors located in the retroperitoneum is shorter than that of the other sites; **(H)** Influence of histopathological grading on the OS of patients; the higher the histopathological grade, the shorter the OS. ANT, adjacent normal tissue; *P < 0.05; ***P < 0.001; ****P < 0.0001.

Univariate analysis indicated that with the AOD value of 0.1950 as the boundary, the low CCNDBP1 protein expression level (P = 0.0016, **Figure 3A**), clinical-stage IV (P < 0.0001, **Figure 3B**), and tumor site (P = 0.0194, **Figure 3C**) were correlated with the short PFS of the patients. Furthermore, in the multivariate Cox regression analysis, the CCNDBP1 protein expression level [P = 0.002, Hazard Ratio (HR) = 0.994, 95% CI: 0.990–0.998] and the clinical staging of the patients (P < 0.001, HR = 8.234, 95% CI: 2.820–24.038) were independent influencing factors of PFS (**Table 2**).

**Table 2:** Univariate and multivariate analysis of the progression-free survival of patients.

Variables	PFS		
	Univariate	Multivariate	
	P-value	HR (95%CI)	P-value
**Expression of CCNDBP1**(Low *vs*. High)	0.0016***	0.994 (0.990–0.998)	0.002**
**AJCC anatomic stage** (Stage II/III *vs*. Stage IV)	<0.0001****	8.234 (2.820–24.038)	<0.001***
**Tumor site (**Retroperitoneum *vs*. Others**)**	0.0194	–	–
**Histologic grade** (G2 *vs*. G3)	0.0544	–	–

PFS, Progressive free survival; HR, Hazard ratio; CI, Confidence interval; AJCC, American Joint Committee on Cancer. **P < 0.01, ***P < 0.001, ****P < 0.0001.

Next, we analyzed the correlation between the CCNDBP1 protein expression level and clinical staging of patients, and determined that the more advanced the clinical stage, the lower the CCNDBP1 expression level in the tumor tissues of the patients (**Figure 3D**). Similarly, the univariate analysis found that the CCNDBP1 expression level (P = 0.0188, **Figure 3E**), AJCC staging (P < 0.0001, **Figure 3F**), tumor site (P = 0.0161, **Figure 3G**), and histological grading (P = 0.0283, **Figure 3H**) were the influencing factors of the OS of the patients, while in the multivariate Cox regression analysis, only the AJCC staging (P < 0.001, HR = 8.550, 95% CI: 2.997–24.391) was the independent hazardous factor that influenced the OS of patients in this group (**Table 3**).

**Table 3:** Univariate and multivariate analysis affecting the overall survival of patients.

Variables	OS		
	Univariate	Multivariate	
	P-value	HR (95%CI)	P-value
**Expression of CCNDBP1**(Low *vs*. High)	0.0188*	–	–
**AJCC anatomic stage** (Stage II/III *vs*. Stage IV)	<0.0001****	8.550 (2.997–24.391)	<0.001***
**Tumor site (**Retroperitoneum *vs*. Others**)**	0.0161*	–	–
**Histologic grade** (G2 *vs*. G3)	0.0283*	–	–

OS, Overall survival; HR, Hazard ratio; CI, Confidence interval; AJCC, American Joint Committee on Cancer. *P < 0.05, ***P < 0.001, ****P < 0.0001.

We used the gray scale to analyze the results of the IHC pictures, and divided the patients into two groups: high (AOD > 0.1950) and low (AOD ≤ 0.1950) CCNDBP1 expression level groups. Both groups were consequently related to the clinicopathological characteristics of the patients (**Table 4**). Interestingly, CCNDBP1 expression had significant differences at the stage M of patients (Chi-square with Yates’ correction P = 0.0261) between the two groups, i.e., the patients with metastasis at the baseline level, the CCNDBP1 expression of tissue samples was lower than that of patients without metastasis.

**Table 4:** Correlation between CCNDBP1 expression and clinicopathological characteristics of patients (N = 46).

Variables	Low expression (N = 21)	High expression (N = 25)	P-value
**Gender**			
Male	11	12	0.7672
Female	10	13	
**Age (years)**	59.57 ± 11.33	57.44 ± 12.63	0.5534
**Tumor size (cm)**	16.70 ± 11.49	14.41 ± 9.49	0.4624
**Tumor site**			
Trunk	1	4	0.2372
Limb	2	5	
Retroperitoneum	18	16	
**Operation times**			
1 time	12	11	0.3745
≥2 times	9	14	
**AJCC anatomic stage**			
Stage II	2	3	0.1730
Stage IIIA	4	10	
Stage IIIB	7	9	
Stage IV	8	3	
**T classification**			
T1	2	3	0.5253
T2	5	9	
T3	6	3	
T4	8	10	
**N classification**			
N0	19	23	0.7319
N1	2	2	
**M classification**			
M0	14	24	0.0261*
M1	7	1	
**Histologic grade**			
G2	8	9	0.8834
G3	13	16	

AJCC, American Joint Committee on Cancer. *P < 0.05.

### CCNDBP1 Significantly Inhibits the Clone Formation, Proliferation, Migration, and Invasion Capacities of LPS853 and LPS510, and Could Accelerate Cell Apoptosis

**Figure 4:**
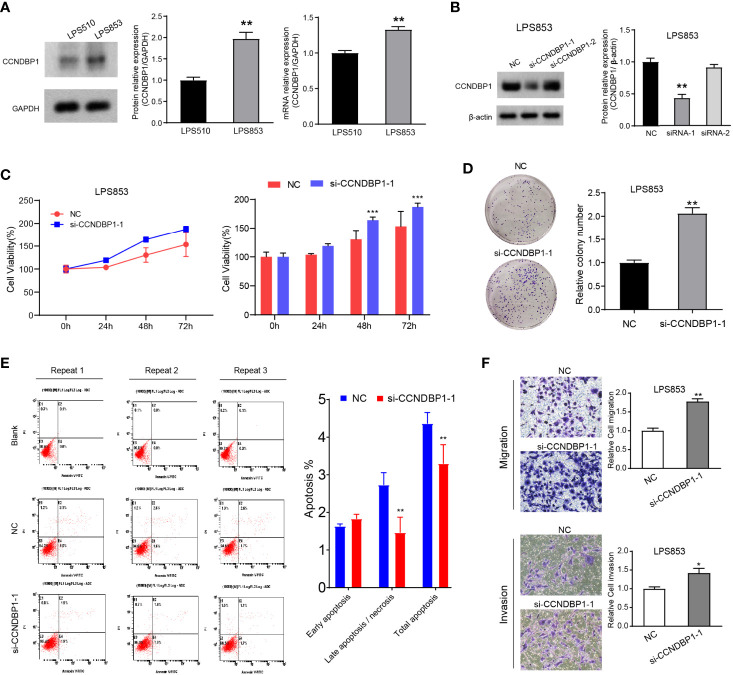
Interfering with the expression of CCNDBP1 could significantly promote the clone formation, proliferation, migration, and invasion capacities of LPS853, and could decrease cell apoptosis. **(A)** LPS853 cells had a higher CCNDBP1 protein and mRNA expression level than LPS510; **(B)** Compared with the NC group, CCNDBP1 protein expression level declined after transfected CCNDBP1 siRNA1, and the difference had a statistical significance; **(C)** CCK8 experiment verified that the cell proliferation capacity was improved after the interference of CCNDBP1 expression in LPS853 and significant difference occurred from the 48 h; **(D)** Clone formation experiment verified that the clone formation capacity was improved after the interference of CCNDBP1 expression in LPS853; **(E)** The total apoptosis rate of LPS853 cells in the CCNDBP1-siRNA1 group was increased to a certain extent in comparison with the NC group, and the difference between the two groups had a statistical significance, mainly because of decrease in late apoptosis and necrosis cells; **(F)** The migration and invasion capacities of LPS853 in the siRNA group were improved in comparison with the NC group; *P < 0.05; **P < 0.01; ***P < 0.001.

The CCNDBP1 protein and mRNA levels in LPS853 cells were higher than those in LPS510 cells (**Figure 4A**), so siRNA interference assays in LPS853 cells and overexpression assays in LPS510 cells were performed.

We knocked down the CCNDBP1 gene with two siRNAs (siRNA-1, siRNA-2) and found that CCNDBP1 was downregulated significantly by siRNA-1, so we used siRNA-1 for the follow-up experiments (**Figure 4B**). First, we specifically used siRNA-1 to interfere with the endogenous cellular expression of CCNDBP1 and conducted clone formation and CCK-8 cell proliferation assays after 48 h. The results indicated that LPS853 cell proliferation (**Figure 4C**, P = 0.0003 at 48 h; P = 0.0004 at 72 h) and clone formation (**Figure 4D**, P = 0.0014) capacities increased significantly after the interference of CCNDBP1 expression. Hence, we studied the influence of the interference of CCNDBP1 expression on the apoptosis of LPS853 cells (**Figure 4E**), and found that at 48 h after siRNA transfection, the total apoptosis rate declined in comparison to that in the untransfected LPS853 cells (P = 0.0046), from 4.37 ± 0.17% to 3.30 ± 0.29%; the decrease was mainly seen in late apoptosis cells (P = 0.0012), from 2.73 ± 0.32% to 1.47 ± 0.40%. Next, we assessed the influence of CCNDBP1 on the metastatic capacity *in vitro*. Transwell assays revealed that the interference of CCNDBP1 increased the migration and invasion capacities of LPS853 cells (**Figure 4F**).

**Figure 5:**
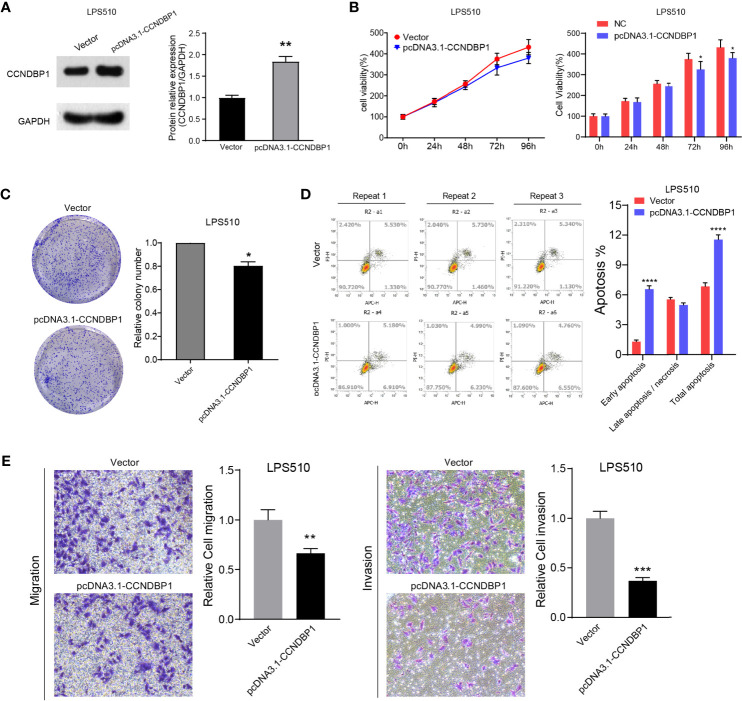
Overexpression of CCNDBP1 could significantly inhabit the clone formation, proliferation, migration, and invasion capacities of LPS510, and could accelerate cell apoptosis. **(A)** Compared with the vector group, CCNDBP1 protein expression level increased after transfected pcDNA3.1-CCNDBP1, and the difference had a statistical significance; **(B)** CCK8 experiment verified that the cell proliferation capacity was inhibited after CCNDBP1 overexpression in LPS510 and a significant difference occurred from the 72 h; **(C)** Clone formation experiment verified that the clone formation capacity was inhibited after CCNDBP1 overexpression in LPS510; **(D)** The total apoptosis rate of LPS510 cells in the pcDNA3.1-CCNDBP1 group was declined in comparison with the vector group, and the difference between the two groups had a statistical significance, mainly because of the increase of the early apoptosis cells; **(E)** The migration and invasion capacities of LPS510 in the pcDNA3.1-CCNDBP1 group were inhibited in comparison with the vector group; *P < 0.05; **P < 0.01; ***P < 0.001; ****P < 0.0001.

We also performed CCNDBP1 overexpression assays by plasmid transfection in LPS510 cells (**Figure 5A**). CCK-8 (**Figure 5B**, P = 0.0436 at 72 h; P = 0.0335 at 96 h) and clone formation assays (P = 0.02, **Figure 5C**) indicated that CCNDBP1 overexpression significantly inhibits LPS510 cell proliferation. In addition, CCNDBP1 overexpression increases the total apoptosis rate of LPS510 (P < 0.0001, **Figure 5D**), from 6.84 ± 0.36% to 11.54 ± 0.48%. However, the increase was mainly in the early apoptosis cells (P < 0.0001), from 1.31 ± 0.17% to 6.56 ± 0.34%. Transwell assays with or without Matrigel indicated that CCNDBP1 overexpression could inhibit the migration (P = 0.0063) and invasion (P = 0.0001) capacities of LPS510 cells (**Figure 5E**).

In summary, CCNDBP1 could significantly inhibit the clone formation and proliferation capacities of DDL, weaken the malignant potential of migration and invasion, and accelerate cell apoptosis *in vitro*. CCNDBP1 could play the role of a tumor suppressor in DDL.

### CCNDBP1 Could Regulate the Malignant Potentials of DDL Cells by Repressing Pathological Epithelial-Mesenchymal Transition

**Figure 6:**
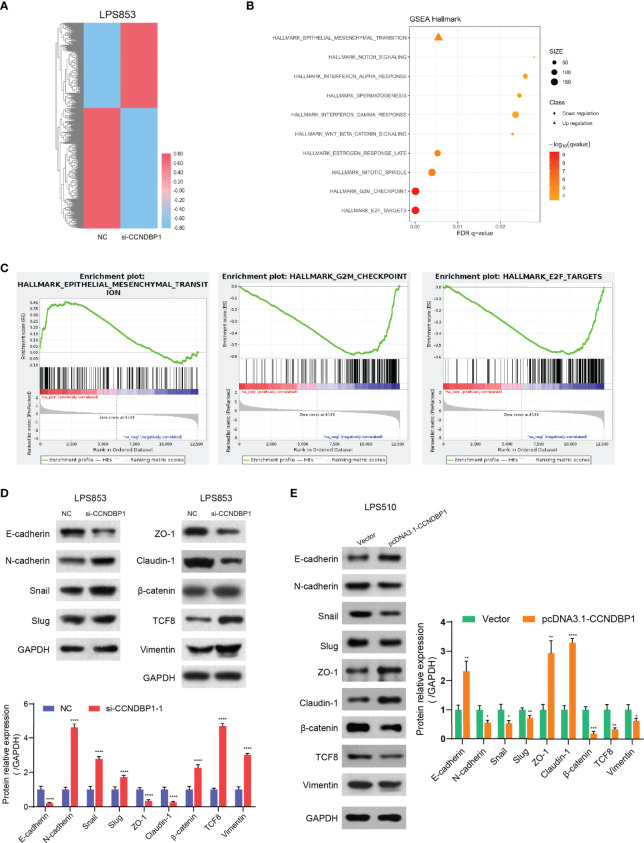
CCNDBP1 could regulate the malignant potentials of DDL cells by repressing the pathological EMT. **(A)** The heatmap of gene expression after the interference of CCNDBP1 expression by siRNA in LPS853 cells. NC as the control, red for upregulation and blue for downregulation; **(B)** Gene function annotation cluster of the GSEA Hallmarks. Clusters with a statistical significance (FDR q < 0.05) were mainly marked; **(C)** The result subset of GSEA, mainly involving the EMT, E2F targets, and G2M checkpoints; **(D)** Based on the Western blot experiments, the expression levels of the EMT-related markers were significantly changed after the interference of CCNDBP1 expression in LPS853 cells. The expressions of E-cadherin, ZO-1, and Claudin-1, proteins related with epithelial cell structures and tight junctions, were apparently downregulated; and the expressions of N-cadherin and Vimentin, markers related with mesenchymal cell status, were apparently upregulated. The expressions of TCF-8, β-catenin, Snail, and Slug, which were transcription factors related with pathological EMT, were apparently upregulated; **(E)** The expression levels of EMT-related markers were significantly changed after CCNDBP1 overexpression in LPS510 cells. The expression of E-cadherin, ZO-1, and Claudin-1 proteins related to epithelial cell structures and tight junctions, was upregulated, while the expression of N-cadherin and Vimentin markers related to the mesenchymal cell status was downregulated. In addition, the expression of TCF-8, b-catenin, Snail, and Slug, transcription factors related to the pathological EMT, were downregulated. GAPDH as the control; *P < 0.05; **P < 0.01; ***P < 0.001; ****P < 0.0001.

To explore the inhibitory effects of CCNDBP1 for DDL cell proliferation and metastasis, we applied RNA-seq to analyze the influence on the gene expression profile changes of LPS853 before and after the interference of *CCNDBP1* expression. The results indicated that *CCNDBP1* gene expression was downregulated by 5.14-fold (5.078/0.987) after siRNA transfection, and 1,290 genes were influenced, including 757 downregulated and 533 upregulated genes (**Figure 6A**). Also, the differential gene enrichment hallmarks influenced by *CCNDBP1* mainly included EMT, targeted E2F, and G2M checkpoints through GSEA (**Figures 6B, C**).

In our current study, we found that DDL was more malignant than WDL and CCNDBP1 play an important role in the migration and invasion of DDL. As EMT (Epithelial-Mesenchymal Transition) was deemed as the key step for tumor cells to obtain the invasion and metastasis capacities (18–20), we speculate that CCNDBP1 may be related to the EMT process. Therefore, we analyzed the expression of key proteins in the EMT process before and after the interference of CCNDBP1 expression. The results indicated that the expression of E-cadherin, ZO-1, and Claudin-1 proteins related with epithelial cell structures and tight junctions was downregulated, while the expression of N-cadherin, Vimentin, and ASMA, markers related to the mesenchymal cell status was upregulated. In addition, the expression of TCF-8, β-catenin, Snail, and Slug, transcription factors related to the pathological EMT, were upregulated (P < 0.0001, **Figure 6D** and **Figure S1A**). When CCNDBP1 is overexpressed in LPS510 cells, we can get the opposite results (**Figure 6E** and **Figure S1A**). Therefore, CCNDBP1 affects the migration and invasion of DDL cells through the pathological EMT process.

### Methylation Degrees of DNA Methylation Sites of CCNDBP1 Influenced its Expression and Prognosis of Dedifferentiated Liposarcoma Patients

DNA methylation is a crucial modification pattern of nucleic acid and can regulate gene expression. To probe the CCNDBP1 expression regulation mechanism preliminarily, we started from DNA methylation, and used theMethSurv database (https://biit.cs.ut.ee/methsurv/) (17) to analyze all the methylation sites of CCNDBP1 (**Table 5**).

**Table 5:** The methylation sites of CCNDBP1.

Composite Element REF	Chromosome	Start	End	CGI_Coordinate	Feature_Type
cg00650309	chr15	43184154	43184155	CGI:chr15:43185318-43185618	N_Shore
cg01678799		43189543	43189544		S_Shelf
cg03626025		43185897	43185898		S_Shore
cg05194114		43185198	43185199		N_Shore
cg07621610		43185503	43185504		Island
cg09296044		43185408	43185409		Island
cg10582045		43185598	43185599		Island
cg12113132		43184725	43184726		N_Shore
cg13892902		43185842	43185843		S_Shore
cg19548922		43191552	43191553		.
cg20581322		43185790	43185791		S_Shore
cg20905760		43185805	43185806		S_Shore
cg21612054		43185747	43185748		S_Shore
cg22184989		43186223	43186224		S_Shore
cg22396798		43185824	43185825		S_Shore
cg23719130		43185418	43185419		Island
cg24184180		43185978	43185979		S_Shore
cg24685109		43184892	43184893		N_Shore
cg26552321		43185257	43185258		N_Shore

**Figure 7:**
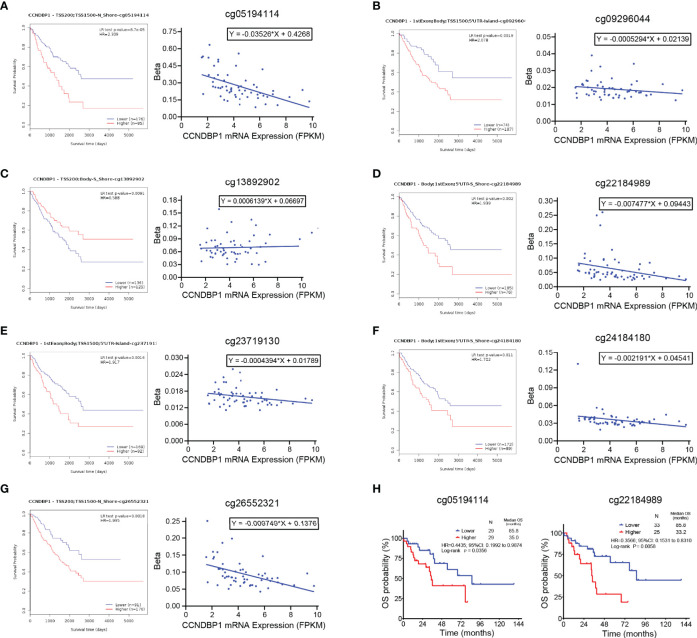
Methylation degrees of DNA methylation sites of CCNDBP1 influenced its expression and prognosis of DDL patients. **(A–G)** 7 DNA methylation sites of CCNDBP1 related with the prognosis of sarcoma patients. Among them, the beta values of **(A)** cg05194114, **(B)** cg09296044, **(D)** cg22184989, **(E)** cg23719130, **(F)** cg24184180, and **(G)** cg26552321 were negatively correlated with CCNDBP1 expression, the larger the beta value, the worse the prognosis; the beta value of cg13892902 **(C)** was positively correlated with CCNDBP1 expression, the larger the beta value, the better the prognosis. **(H)** The survival curve of DNA methylation degrees of cg05194114 (with the beta value of 0.2389 as the boundary) and cg22184989 (with the beta value of 0.04997 as the boundary) influenced the OS of DDL patients in TCGA database, the higher the methylation degrees of these two sites, the worse the prognosis of DDL patients.

Next, the correlations among these sites with the OS of sarcoma patients was analyzed, and the following sites were found to be associated with the prognosis of patients: cg05194114 (P = 0.000087, **Figure 7A**), cg09296044 (P = 0.0019, **Figure 7B**), cg13892902 (P = 0.0091, **Figure 7C**), cg22184989 (P = 0.002, **Figure 7D**), cg23719130 (P = 0.0016, **Figure 7E**), cg24184180 (P = 0.011, **Figure 7F**), and cg26552321 (P = 0.0018, **Figure 7G**). In addition, linear regression analysis indicated that the DNA methylation degree of cg13892902 was positively correlated with CCNDBP1 expression, and the remaining methylation sites were negatively correlated (**Figures 7A–G**). The results above further revealed that the higher the expression of CCNDBP1, the better the prognosis of patients.

Furthermore, we screened the DNA methylation sites related to the prognosis of DDL patients and identified that the DNA methylation degrees of cg05194114 (P = 0.0356) and cg22184989 (P = 0.0058) were associated with the OS of DDL patients (**Figure 7H**). Thus, these two sites might be the primary sites of CCNDBP1 expression regulation in DDL patients.

## Discussion

The morphology of DDL is the WDL area and non-adipose-derived sarcoma area formed by a sudden transition, and DDL is composed of these two tumor components (21). Thus, it is not difficult to understand that DDL has the same cellular and molecular genetics features as WDL. About 10% (3, 4) of the intermediate (local invasion) WDL cases have a malignant transformation into DDL. The four DDL tissue specimens in this study were matched with four WDL tissue specimens according to the baseline characteristics, such as gender, age, and recurrence, before high-throughput sequencing was conducted. Genes that did not cause WDL to obtain local invasion and distant metastasis capacities were excluded to a great extent, providing reliable subsequent results. To the best of our knowledge, there is still no ideal biomarker for the prediction of DDL patient prognosis in the clinical practice. TCGA database recorded the clinical follow-up data, and transcriptome sequencing results of 58 DDL patients and genes related to prognosis could be obtained through analysis. These genes were integrated with our sequencing results and verified with the GEPIA database in soft tissue sarcomas. Finally, we identified *CCNDBP1*, the gene with a prognostic predictive value.

CCNDBP1 is expressed mainly in terminally differentiated tissues and might play a critical role in controlling cell differentiation and proliferation (22). The overexpressed CCNDBP1 could inhibit the proliferation of breast cancer cell line MCF-7 (23) and NSCLC cell line H1299 (24), while the decrease in CCNDBP1 stability could accelerate the proliferation, migration, and invasion of lung cancer cell line and gastric cancer cell line (25). Some animal experiments indicated that the overexpression of CCNDBP1 in transgenic mice inhibited liver tumors induced by diethylnitrosamine (26). The mice with livers lacking CCNDBP1 expression had an early development of hepatocellular carcinoma (27). Moreover, in some progressive or metastatic cancer tissues (including breast cancer, prostatic cancer, and colon cancer), CCNDBP1 expression declined (28). In breast cancer, the decrease in CCNDBP1 expression was correlated with the poor prognosis of patients (29).

In DDL, the prognostic predictive value of CCNDBP1 has not been reported. The current study, for the first time, confirmed that the correlation of a low CCNDBP1 protein expression level with the poor prognosis and expression level of DDL patients was an independent prognosis influencing factor of the PFS of patients; it was associated with the clinical staging and could provide a hint about the potential occurrence of distant metastasis. The ROC curve indicated that the sensitivity to predict the progression of DDL patients was 90.91%, but the specificity was poor at only 57.14%, with the cutoff of the AOD value for CCNDBP1 IHC staining was >0.1950. Previous studies reported that CCNDBP1 was correlated with the occurrence and development of breast cancer, colon cancer, liver cancer, non-small cell lung cancer, osteosarcoma, and gastric cancer (9, 23–29). Therefore, the expression of CCNDBP1 might not be tumor-specific. In order to further verify the role of CCNDBP1 as a tumor suppressor in DDL, we conducted *in vitro* experiments and found that CCNDBP1 significantly inhibited the clone formation, proliferation, migration, and invasion capacities of DDL cell lines. In addition, CCNDBP1 accelerated cell apoptosis. In summary, CCNDBP1 is a major tumor suppressor of DDL and could be used as a prognostic marker for the prediction of DDL metastasis.

In this study, we analyzed hallmarks enriched by differential genes of DDL cell line LPS853 before and after the siRNA interference of CCNDBP1 expression, as assessed by GSEA, and found that the biological processes with the lowest false discovery rate (FDR) q-value included the EMT, targeted E2F, and G2M checkpoints. The influence of CCNDBP1 on the Rb/E2F signaling pathways and G2M checkpoint regulation has been reported in osteosarcoma (9). EMT is a key process of tumor dedifferentiation to obtain invasion, migration, and other malignant potentials (18–20). During this process, epithelial cells acquire mesenchymal fibroblast-like properties that show the enhancement of migration capacity and invasiveness (19). Therefore, the regulatory effect of CCNDBP1 on the EMT process is the focus of this study.

EMT involves changes to cell phenotypes, and this process is regulated by complex signaling pathways and a network of EMT transcription factors (20, 30). Among these, E-cadherin is regarded as the invasion and growth activity inhibition protein in several epithelial carcinoma cells (31–33). In cancer cells, the loss of E-cadherin often results in the metastatic spread of tumors and the activation of multiple types of EMT transcription factors (34). In addition to the loss of E-cadherin in cancer cells, the expression of N-cadherin is often upregulated, and this conversion among cadherin expressions is called “cadherin conversion” (30, 35). N-cadherin can be used as an indicator of the ongoing EMT process, and its expression is related to the occurrence and development of various cancers (36–39). Vimentin is an intermediate fiber of mesenchymal cells and a critical mesenchymal tissue marker, which could be found at the early developmental stage (40). It also regulates cell adhesion and movement through its phosphorylation (soluble form) and dephosphorylation (insoluble form), and the expression level is associated with an increased risk of tumor metastasis (41–43). EMT is marked by the upregulation of N-cadherin, Vimentin, and ASMA, and downregulation of E-cadherin, which plays a critical role in the process of tumor metastasis (30, 44, 45). In this study, after interfering with the CCNDBP1 expression, the expression of these four key EMT-related markers altered significantly, which is consistent with the changes mentioned above, indicating that CCNDBP1 could inhibit the process of EMT from weakening the invasion and migration capacities of DDL cells.

During EMT, E-cadherin rupture resulted in an unstable adherent junction, and consequently releasing β-catenin, an activating transcription factor for cell proliferation (46, 47). β-catenin is a key downstream effector of the Wnt signal transduction pathway (47). The increase in the total amount of β-catenin protein in cells could activate the Wnt-targeted genes (48), and the activation of the Wnt/β-catenin pathway is correlated with stemness and the early formation of tumor cells (49). β-catenin can also participate in the activation of EMT *via* the Slug protein in breast cancer (50). Slug is a member of the Snail family of zinc-finger transcription factors and a type of extensively expressed transcription inhibition protein (51). Snail/Slug binds with the E-cadherin promoter region to inhibit its transcription, thereby reducing cell-to-cell adhesion and accelerating the migration, invasion, and metastasis of cancer cells (52, 53). Snail/Slug are downstream effectors of the TGF-β and MAPK signaling pathways. Some studies have indicated that the Snail/Slug signaling pathway accelerates the EMT process, effectuating the proliferation, invasion, and metastasis of gastric cancer cells (54). Both ZO-1 and Claudin-1 mutations induce EMT (55). Both proteins are both tight junction-related proteins, and their expression is decreased in the process of tumor formation, which has a correlation with the absence of the tumor differentiation capacity (56). Previous studies have shown that the decrease in the expression of ZO-1 and Claudin-1 accelerates the invasion and migration of pancreatic cancer *via* a ZEB1-dependent transcription (56). ZEB1, namely TCF-8, is a member of the ZEB protein family, a transcription factor-containing zinc finger, and homeodomain that can inhibit the expression of E-cadherin and play a key role in tumor progression-related pathological EMT process (57). Reportedly, ZEB1 can regulate the Wnt/β-catenin signaling pathway (58, 59) and the TGF-β-dependent EMT process (60). EMT is a complicated process with multiple different signaling pathways coordinates (30). In this study, the expression of β-catenin, ZEB1, Slug, and Slug were upregulated in LPS853 cell line after the interference of CCNDBP1 expression, which might activate the Wnt/β-catenin, Snail/Slug, TGF-β, and MAPK signaling pathways to induce the occurrence of pathological EMT and enhance the malignancy degree of DDL.

The TCGA database disclosed the DNA methylation information of 58 cases of specimens from patients with DDL, and the prognosis of patients could be predicted based on the gene methylation sites. To preliminarily probe whether CCNDBP1 was methylation-regulated in sarcoma patients, we screened the methylation positions of genes with prognosis values in sarcoma *via* the MethSurv database, downloaded the sequencing data of these methylation sites in 58 DDL patients and plotted the survival curves. We found that the methylation degrees of cg05194114 and cg22184989, two CCNDBP1 methylation sites, were correlated with the OS of DDL patients. DNA methylation analysis is not limited to tissue samples but can be extended to almost any type of body fluid. For example, circulating tumor DNA (ctDNA) in plasma is the carcinogenic ingredient of cell-free DNA (cfDNA), which can provide tumor mutation and epigenetic inheritance information for prognosis prediction. Also, ctDNA is very easily detected by the non-invasive or minimally invasive technology, and, hence, is valuable for tumor tissues on which surgery or biopsy cannot be conducted (61). Currently, many studies indicated that ctDNA could be used as a potential biomarker for the prediction of tumor patient prognosis (62–65). Therefore, subsequent studies can use ctDNA as a breakthrough to design the relevant primers to detect the methylation degrees of cg05194114 and cg22184989 in the plasma ctDNA of patients with DDL for the prediction of the prognosis.

Nevertheless, the present study has some limitations. On the one hand, limited by conditions, we could not conduct *in vivo* experiments to further confirm the anti-cancer effect of CCNDBP1 in DDL, thereby necessitating follow-up studies. On the other hand, the regulatory mechanism of this gene was not investigated, and only data from public databases were used for the analysis in methylation and had not conducted corresponding experiments for verification. Taken together, the abovementioned defects could be directions of subsequent research to provide more sufficient evidence for the function of CCNDBP1 as the prognostic prognosis marker of DDL patients.

## Conclusions

**Figure 8:**
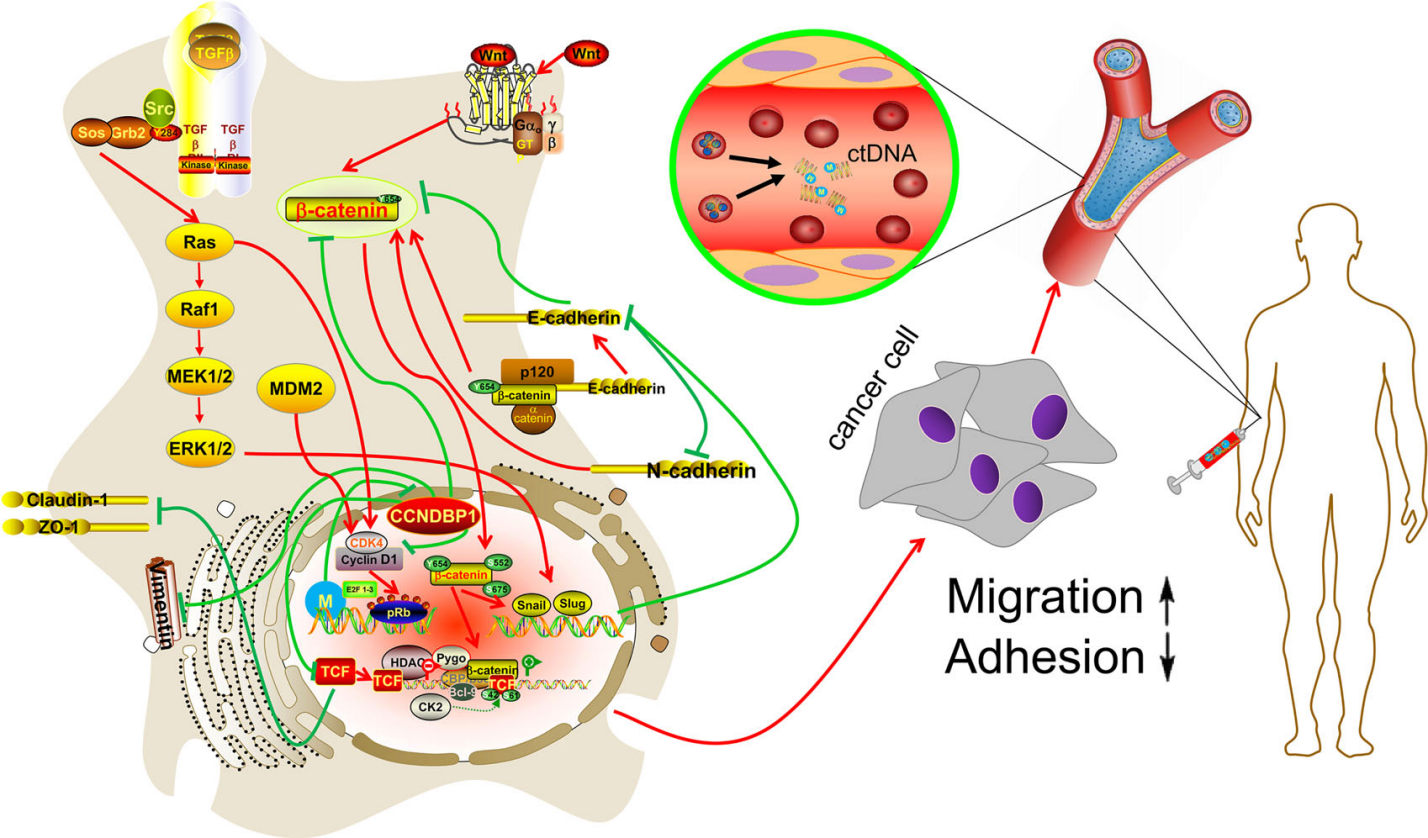
Hypothetical model. CCNDBP1 could inhibit the migration and invasion capacities of DDL by regulating the EMT process, which could be conducted by repressing a variety of signaling pathways, such as the Wnt/β-catenin, Snail/Slug, TGF-β, and MAPK. In addition, the methylation site might be the regulation of CCNDBP1 gene expression in DDL, which could drive future research, including the detection of the methylation level of CCNDBP1 in plasma ctDNA by liquid biopsy.

In summary, we first reported the potential value of CCNDBP1 for the prediction of the prognosis in patients with DDL, and its low expression level was associated with a poor prognosis. CCNDBP1 is an independent prognostic factor for the PFS in our cohort, mainly related to metastatic tumor patients at baseline characteristics. Furthermore, we conducted *in vitro* experiments and verified that CCNDBP1 could inhibit the clone formation, proliferation, migration, and invasion capacities of DDL and promote the apoptosis by regulating the EMT process, which could be conducted by repressing a variety of signaling pathways, such as the Wnt/β–catenin, Snail/Slug, TGF–β, and MAPK. In addition, the expression of CCNDBP1 could be regulated *via* its methylation level and the primary regulation sites in patients with DDL were cg05194114 and cg22184989, which are specifically manifested by the low degree of methylation and high expression of CCNDBP1 that improved patient prognosis. These two methylation sites might be the primary sites for the regulation of *CCNDBP1* gene expression in DDL, which could drive future research, including the detection of the methylation level of CCNDBP1 in plasma ctDNA by liquid biopsy (**Figure 8**).

## Data Availability Statement

The original contributions presented in the study are included in the article/**Supplementary Material**, and the RNAseq data presented in the study are deposited in the GEO repository, accession number GSE184199 (https://www.ncbi.nlm.nih.gov/geo/query/acc.cgi?acc=GSE184199). Further inquiries can be directed to the corresponding author.

## Ethics Statement

The studies involving human participants were reviewed and approved by the Ethics Committee of Fudan University Shanghai Cancer Center. The patients/participants provided their written informed consent to participate in this study.

## Author Contributions

Conceptualization: WY, YL, and CW. Data curation: LY, ZW, WS, PL, YC, and SC. Formal analysis: LY, ZW, YC, and SC. Methodology: LY, PL, WS, YC, WY, YL, and CW. Software: LY, ZW, PL, YC, YL, and CW. Supervision: WY, YL, and CW. Validation: ZW, WS, PL, YC, YL, and CW. Visualization: LY, SC, WY, YL, and CW. Writing—original draft: LY. Writing—review and editing: WY, YL, and CW. All authors contributed to the article and approved the submitted version.

## Conflict of Interest

The authors declare that the research was conducted in the absence of any commercial or financial relationships that could be construed as a potential conflict of interest.

## Publisher’s Note

All claims expressed in this article are solely those of the authors and do not necessarily represent those of their affiliated organizations, or those of the publisher, the editors and the reviewers. Any product that may be evaluated in this article, or claim that may be made by its manufacturer, is not guaranteed or endorsed by the publisher.
