# Supplementary material for: CCNDBP1, a Prognostic Marker Regulated by DNA Methylation, Inhibits Aggressive Behavior in Dedifferentiated Liposarcoma via Repressing Epithelial Mesenchymal Transition
Source: Front Oncol. 2021 Sep 22;11:687012. doi: 10.3389/fonc.2021.687012 (PMC8493074; doi:10.3389/fonc.2021.687012)
Supplement: Supplementary file 1 [file DataSheet_1.doc]

Supplementary Material

## Supplementary Tables

**Table S1.** Primer sequences for q-PCR, siRNA and Oligos for plasmid construction

| **Primer name** | **Primer sequence (5 '→ 3')** |
| --- | --- |
| Primers for qPCR | |
| h-CCNDBP1-F | TCAGCCCTAGTGTGGATGATT |
| h-CCNDBP1-R | GTTTCGCAGAATTGATTCGCAC |
| h-GAPDH-F | GGAGCGAGATCCCTCCAAAAT |
| h-GAPDH-R | GGCTGTTGTCATACTTCTCATGG |
| siRNAs | |
| CCNDBP1-siRNA1 | CCCAGAAGTTCTGTGAACAAGTCCA |
| CCNDBP1-siRNA2 | GCAGATGCCTCAGATACCAAGAGAT |
| Oligos for plasmid construction | |
| **CCNDBP1-F** | CTCGGATCCGCCACCatggcgagcgcaactgcacc |
| **CCNDBP1-R** | CCCTCTAGACTCGAGtaattcaagttcactctgag |

**Table S2.** Clinical information of 4 cases of well-differentiated liposarcoma (WDL) and matched 4 cases of dedifferentiated liposarcoma (DDL)

| **Sample ID** | **Age** | **Gender** | **Pathological types** | **Primary/Recurrence** | **Size (mm)** |
| --- | --- | --- | --- | --- | --- |
| R1 | 79 | Male | WDL | Recurrence | 128 |
| R2 | 45 | Female | WDL | Primary | 58 |
| R3 | 67 | Female | WDL | Recurrence | 50 |
| R4 | 64 | Male | WDL | Primary | 85 |
| R5 | 52 | Female | DDL | Primary | 98 |
| R6 | 65 | Female | DDL | Recurrence | 120 |
| R7 | 75 | Male | DDL | Recurrence | 80 |
| R8 | 57 | Male | DDL | Primary | 47 |

**Table S3.** Results of RNA concentration measurement of eight tissue samples used for sequencing

| **Sample ID** | **Pathological types** | **Nucleic Acid （ng/µl）** | **A260 (Abs)** | **A280 (Abs)** | **260/280** | **260/230** |
| --- | --- | --- | --- | --- | --- | --- |
| R1 | WDL | 424.2 | 10.605 | 5.214 | 2.03 | 1.94 |
| R2 | WDL | 147 | 3.676 | 1.784 | 2.06 | 1.99 |
| R3 | WDL | 297.2 | 7.43 | 3.611 | 2.06 | 1.98 |
| R4 | WDL | 74.6 | 1.864 | 0.888 | 2.10 | 1.99 |
| R5 | DDL | 416 | 10.4 | 5.139 | 2.02 | 2.10 |
| R6 | DDL | 200.1 | 5.002 | 2.409 | 2.08 | 2.07 |
| R7 | DDL | 164.6 | 4.114 | 1.964 | 2.09 | 1.90 |
| R8 | DDL | 197.5 | 4.937 | 2.499 | 1.98 | 1.92 |

## Supplementary figure


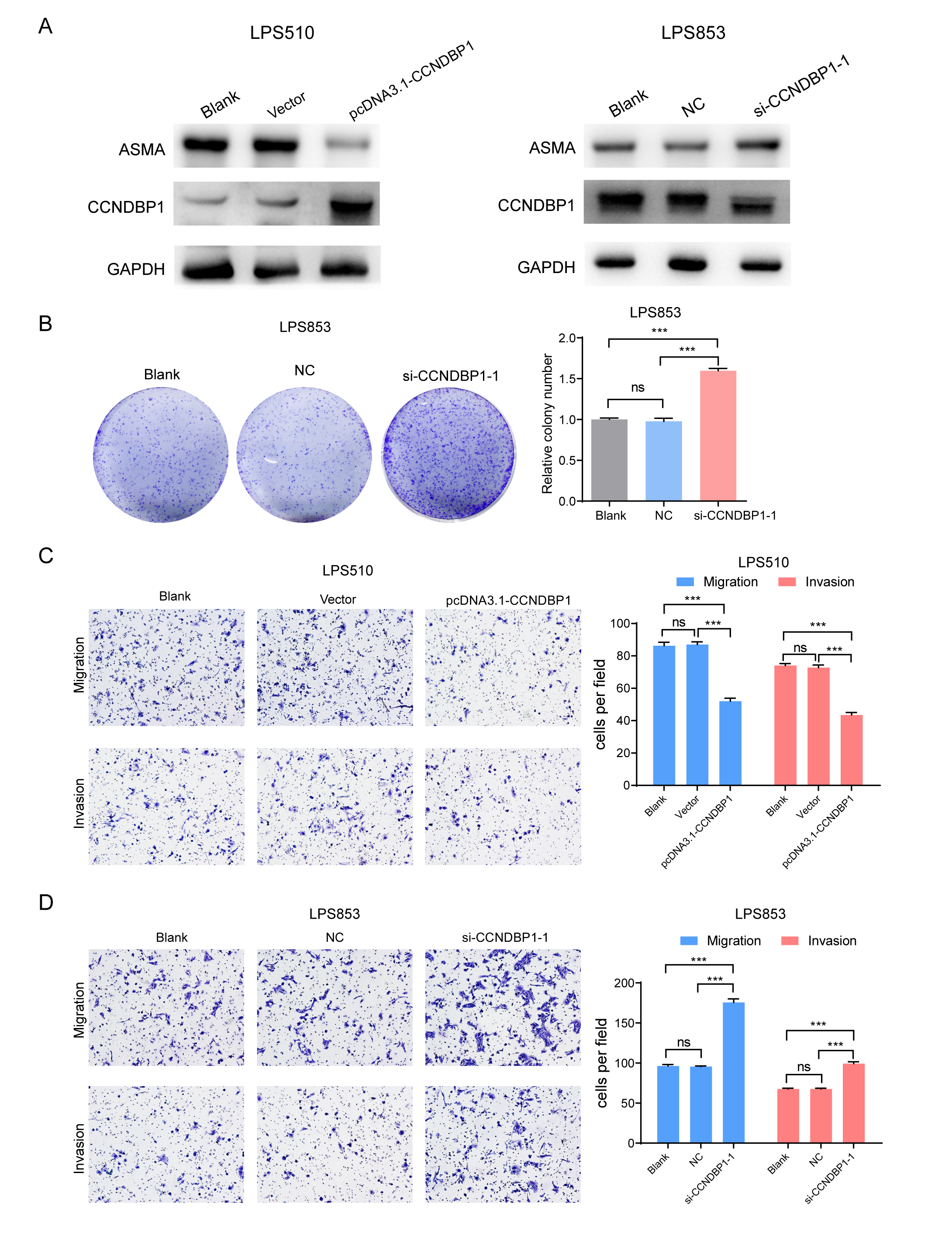


Figure S1. Effect of CCNDBP1 on the expression of ASMA and DDL cell proliferation, migration and invasion. A. ASMA protein levels were determined by Western blotting following CCNDBP1 overexpression or siRNA transfection. B. Colony formation assays were performed to observe the effects of CCNDBP1 knockdown on cell proliferation in LPS853. C.Transwell migration and invasion assays were performed in LPS510 after transfection CCNDBP1 plasmid pcDNA3.1-CCNDBP1 or empty vector. Empty cells were the control group (Blank). D. Transwell migration and invasion assays in LPS853 transfected with CCNDBP1 siRNA or NC. Statistical analysis was performed with Student’s t-test.*P<.05; **P<.01; ***P<.001. Data represent mean ± SEM
